# Anticancer Activity of the Marine-Derived Compound Bryostatin 1: Preclinical and Clinical Evaluation

**DOI:** 10.3390/ijms26167765

**Published:** 2025-08-11

**Authors:** Tomasz Kowalczyk, Marek Staszewski, Magdalena Markowicz-Piasecka, Joanna Sikora, Catarina Amaro, Laurent Picot, Przemysław Sitarek

**Affiliations:** 1 Department of Molecular Biotechnology and Genetics, Faculty of Biology and Environmental Protection, University of Lodz, Banacha 12/16, 90-237 Lodz, Poland; 2 Department of Synthesis and Technology of Drugs, Medical University of Lodz, Muszynskiego 1, 90-151 Lodz, Poland; marek.staszewski@umed.lodz.pl; 3 Department of Applied Pharmacy, Medical University of Lodz, Muszynskiego 1, 90-151 Lodz, Poland; magdalena.markowicz@umed.lodz.pl; 4 Department of Bioinorganic Chemistry, Medical University of Lodz, Muszynskiego 1, 90-151 Lodz, Poland; joanna.sikora@umed.lodz.pl; 5 Students Research Group (Erasmus Student), Department of Medical Biology, Medical University of Lodz, 90-151 Lodz, Poland; catari.amaro@gmail.com; 6 Littoral Environnement et Sociétés UMRi CNRS 7266 LIENSs, La Rochelle Université, 17042 La Rochelle, France; laurent.picot@univ-lr.fr; 7 Department of Medical Biology, Medical University of Lodz, Muszynskiego 1, 90-151 Lodz, Poland

**Keywords:** *Bugula neritina*, Bryostatin 1, modulator of protein kinase C, anticancer effect, clinical trials

## Abstract

Bryostatin 1, a natural macrolide isolated from *Bugula neritina*, is a potent modulator of protein kinase C (PKC) isoforms with promising anticancer properties. In numerous in vitro studies, bryostatin 1 has been shown to inhibit tumor cell proliferation and induce differentiation and apoptotic cell death in a wide range of cell lines, including leukemia, lymphoma, glioma, and solid tumors such as ovarian and breast cancer. Its antitumor activity, both as monotherapy and in combination with conventional chemotherapy, has been confirmed in in vivo models, where synergistic effects have been observed, including sensitization of tumor cells to cytostatic agents. Despite promising preclinical findings, phase I and II clinical trials have not yielded the expected results, suggesting limited efficacy of the macrolide as a single agent with a relatively favorable safety profile. Current research directions focus on optimizing dosing regimens, combining bryostatin 1 with other anticancer drugs and identifying predictive biomarkers of response. This article reviews the current state of knowledge on the anticancer effects of bryostatin 1, analyzing available data from in vitro, in vivo, and clinical trials and discussing potential directions for further translational research.

## 1. Introduction

According to the World Health Organization, cancer is one of the leading causes of mortality globally, responsible for nearly ten million deaths in 2020, equivalent to approximately one in every six deaths [1]. In 2022, an estimated 20 million new cancer cases were diagnosed, while the five-year prevalence exceeded 53 million individuals. The most frequently affected anatomical sites include the lungs, breast, colorectum, prostate, stomach, and liver [2]. These high figures decrease if the cancer is detected early and treated effectively. Standard cancer treatments include surgery, radiotherapy, and chemotherapy [3]. Small molecule-targeted therapy and chemotherapy are two therapeutic approaches applied to treat cancer with the use of chemical compounds such as cisplatin, doxorubicin and paclitaxel. Despite their efficacy, chemotherapeutic drugs can cause various adverse reactions in normal cells, ranging from nausea to neuropathy and myelosuppression. Furthermore, they are associated with multidrug resistance, a phenomenon accounting for more than 90% of deaths among cancer patients undergoing chemotherapy [4].

Owing to their structural diversity, selective cytotoxicity and generally favorable safety profiles, natural products have been increasingly used as anticancer agents as a well-recognized therapeutic strategy. These types of compounds, used since the ancient times, have been an incomparable source of anticancer drugs. Although the historical record of natural products is strong, there is a need to investigate and confirm their efficiency as anticancer drugs and determine the potential of natural products as an important source of future therapeutic agents. Since many anticancer target-specific medicines have failed to provide successful results, natural products with multi-target characteristics should be investigated to achieve better outcomes. A thorough analysis of all their properties may lead to developing promising novel compounds for cancer treatment and constitutes an important area of research [5]. Substances of marine origin, known as “marine drugs”, are playing an increasingly important role in research into modern cancer therapies. They are characterized by unique chemical structures and potent biological properties that are often not found in terrestrial compounds. Their uniqueness mainly results from several factors, including different mechanisms of action, high selectivity or overcoming drug resistance. Examples of approved marine drugs include trabectedin (trade name: Yondelis, from tunicates), eribulin (trade names: Halaven, Mevlyq, from sea sponges) or plitidepsin (trade name: Aplidin), used to treat plasmocytic myeloma. Research into marine drugs has been developing rapidly, opening up new possibilities in cancer treatment [6,7,8,9,10].

One of these potential active compounds is Bryostatin 1, a natural marine metabolite that was first isolated from the bryozoan *Bugula neritina* in 1968, a small marine invertebrate with a worldwide distribution, living in North and South America, the Mediterranean, and Australia [11,12,13,14]. When *B. neritina* develops embryos, bryostatins are produced due to a symbiotic relationship with the bacteria *Candidatus Endobugula sertula*, which covers them as protection against predators after release into the outside environment [13,14]. More than a decade later, Bryostatin 1 structure was finally characterized in 1982, being recognized as a macrocyclic lactone. This compound is a very promising lead compound because of its potent biological activity against various human diseases [11]. Bryostatin 1 is mostly known as a powerful protein kinase C (PKC) agonist, and thus it can induce cell differentiation, regulate apoptosis, inhibit tumor invasion, affect the activity of certain cell cycle regulatory proteins and modulate the multidrug resistance gene [15,16]. The extensive range of interactions unlocked by bryostatin 1 makes this marine compound a promising anticancer agent, while also highlighting its therapeutic potential for Alzheimer’s disease and, possibly, human immunodeficiency virus (HIV) treatment [17]. In this article, we will review clinical trials, in vitro and in vivo assays to discuss both the clinical potential of bryostatin 1 as an anticancer drug and its mechanisms of action.

## 2. Marine Compounds

The interest in studies on marine-derived compounds has increased in recent years since they show promising activity in diverse sectors, such as the food and cosmetic industries and medicine. In the latter, their potential use as anticancer agents should be emphasized [18,19].

One of the first examples given was Trabectedin, commercially known as Yondelis, which is a chemotherapy agent approved for the treatment of advanced soft tissue sarcoma. It originated from Carribean tunicate *Ecteinascidia turbinata* [20], a colonial species growing in dense clusters of zooids, i.e., multicellular single animals, in a cylindrical form that enlarges gradually towards the end [21]. As for its mechanism of action, Trabectedin can affect key cell biology processes both in tumor cells and its microenvironment [22]. This compound has two rings that bind covalently to the minor groove of DNA and a third ring that binds to the nearby nuclear proteins [20]. Thus, it forms adducts in the minor groove and, consequently, breaks in the template, triggering various transcription factors involved in cell proliferation, via the transcription-coupled nucleotide excision repair system. The cells are then arrested in the G2 phase, rendering those in the G1 phase highly sensitive to this effect. Trabectedin can also inhibit overexpression of the gene responsible for the cells that develop resistance to cancer drugs, known as multidrug resistance protein 1 (MDR-1). Additionally, it reduces the number of tumor-associated macrophages accounting for the release of growth factors, cytokines, and chemokines that promote inflammation and neoangiogenesis while simultaneously inhibiting the secretion of the same cytokines and chemokines. This compound can also reduce myeloid-derived suppressor cells and induce apoptosis exclusively in monocytes and macrophages via activation of caspase-8. Due to its wide range of action, Trabectedin has been studied as a potential agent to be used in the treatment of other types of cancer [20,23].

The second example is Eribulin, an antineoplastic agent that binds to microtubules and inhibits their polymerization, which results in mitotic arrest and cell death by apoptosis. It differs from other antitubulin agents by not affecting the shortening phase of microtubule dynamics and leading to the formation of nonproductive tubulin aggregates, and thus lowering intracellular concentrations of free tubulin. In turn, all this leads to inhibition of mitotic spindle formation [24]. Its origin dates back to 1985 when a derivative called halichondrin B was isolated from the species *Halichondria okadai*, a Japanese marine sponge [25]. Although it showed strong anticancer activity, it was hardly accessible, therefore it was difficult to conduct research focused on this compound. Due to its potential, in 1998, a synthetic macrocyclic ketone analogue, Eribulin, was developed, thus providing easier, sustainable and cost-effective access [26]. Currently, it is approved for the treatment of metastatic breast cancer in heavily pretreated patients, although as shown in various trials, it has also proved to be highly effective in the treatment of other cancer lines [27].

Lastly, Plitidepsin is a peptide, originally isolated from *Aplidium albicans*, a Mediterranean marine tunicate, known as sea-squirts, a primitive invertebrate marine animal. Nowadays, it is synthetically produced and commercialized under the trade name Aplidin [28,29]. It shows antineoplastic activity by connecting to the elongation factor 1A2, which is overexpressed in human tumors and favors tumor cell proliferation while inhibiting apoptosis. By targeting this molecule, Plitidepsin can mediate the tumor activity. This compound can also induce early oxidative stress and sustained activation of p38 mitogen-activated protein kinases, which all leads to apoptosis via caspases. Other benefits of this agent include antiangiogenic effects by blocking the secretion of the angiogenic factor vascular endothelial growth factor (VEGF), which indicates it can affect tumor angiogenesis. Due to its activity against cells that stimulate cancer progression, it also modifies the tumor microenvironment [28,30]. Plitidepsin has been hardly studied alone or in combination with other agents, and, although in numerous trials it has shown diverse effects on various types of cancer, it was rejected by the European Medicines Agency (EMA) and is approved for use only in Australia in patients with refractory multiple myeloma [28,31].

There are other marine-derived drugs that are equally interesting. For example, Cytarabine was the first marine-derived clinically used drug, primarily used to treat non-lymphocytic and lymphocytic leukemia. It is an antineoplastic agent that inhibits DNA synthesis by acting as a pyrimidine nucleoside analogue. It is constituted by the nucleosides spongothymidine and spongouridine derived from the *Tethya crypta*, a large, shallow-water sponge found in the Caribbean Sea [32,33,34]. These nucleosides are also the inspiration for Vidarabine, a synthetic analogue of spongouridine antibiotic, with improved antiviral activity, used for the treatment of varicella, herpes zoster and simplex [35,36]. Similarly, Ziconotide is also a synthetic drug with marine origins, as it is the synthetic form of a peptide that constitutes the venom of the sea snail *Conus magus*, which can even be dangerous to humans [37,38]. Ziconotide is classified as an N-type calcium channel antagonist, used in the treatment of severe chronic pain, particularly in cases where conventional therapies are neither adequate nor effective [39].

Although all of these compounds are promising therapeutic agents, bryostatins show similar promise in terms of interest and activity, being one of the most vastly studied natural marine products [40]. Bryostatins are antineoplastic agents that modulate protein kinase C and its isoenzymes. They all share a common macrolactone core with three tetrahydropyran rings. They distinguish themselves by their substituents at C-7 and C-20 positions, with the last one connected or not to a 2,4-octadienoate moiety, and by the presence of a γ-lactone ring in the C-19 to C-23 tetrahydropyran ring [13].

Bryostatins derive from the marine species, the *Bugula neritina*, a bush-like, calcified aquatic invertebrate animal. This colonial bryozoan is usually found in grass beds, oyster and coral reefs, marinas and docks and can live in temperatures ranging from 2.2 to 30 °C [14]. It comprises individual zooids with tentacles for capturing nourishment, in addition to a funicular system that distributes nutrients to feeding zooids, non-feeding zoids and ovicells, which chambers where fertilized eggs develop into embryos. In this phase of life, bryostatins are produced and delivered as a coating onto the embryos, which, when mature, are released to the outside as non-feeding cells, where they settle on a substrate, before developing asexually to form a new colony. The coating of bryostatins remains on the larvae, previously embryo, physically protecting the cells until they evolve and synthesize a chitin exoskeleton. If a predator consumes the larvae, the bryostatins allow the larvae to be regurgitated and continue to develop [13]. Thus, bryostatins act like shields protecting the *Bugula neritina* life cycle, and it is hypothesized that they are synthesized by a symbiotic relationship between this bryozoan and *Candidatus Endobugula sertula*, a proteobacteria [13,15]. This is because when *B. neritina* has a reduction or lack of *E. sertula*, they show lower levels or absence of bryostatins, respectively. Additionally, colonies that developed from antibiotic-treated larvae also have reduced levels of bryostatins. Consequently, specific populations of the *B. neritina*, with various changes in the *Candidatus Endobugula sertula* gene, tend to have different levels and types of bryostatins. There are two types of *Bugula neritina* that differ in their mitochondrial COI sequence, i.e., type D, containing strain D of *E. sertula* and bryostatins of chemotype O (bryostatins 1 to 3, 12 and 15), and type S that contains type S of the bacteria and chemotype M, producing bryostatins from 4 to 9. These different lineages of *B. neritina* vary in terms of environment factors. Type S is predominant on boat docks and hulls, and can be transported worldwide due to its settlement on boat hulls. The distribution of type D is more restricted since it relies on larval dispersal and is not able to establish a major population on the pier [41].

Unfortunately, *E. sertula* has remained resistant to laboratory culture and direct fermentation, hindering research advances [42]. Additionally, bryostatins are scarce in nature since they are present in small amounts in marine species. To address this issue, several alternative approaches have been explored. One of them is the mariculture of *Bugula neritina*, which has been successfully achieved but not yet implemented on a commercial scale. Another strategy involves the total synthesis of naturally occurring bryostatins, which, although steadily improving, remains impractical for industrial production. A third approach focuses on the synthesis of simplified structural analogues that are easier to produce synthetically and retain biological activity. However, replicating the full therapeutic effects of the original compounds remains a significant challenge [13,42,43].

Around 21 bryostatins were obtained naturally, exhibiting, apart from enhancement of memory and cognition, antineoplastic and synergistic chemotherapeutic activity [30]. Some of them are being studied, and their total synthesis is already known, like in the case of bryostatin 2, 3 and 7 [44,45]. However, only bryostatin 1 has received so much interest, possibly due to being the first one isolated. It can be obtained in larger quantities and shows an interesting pharmaceutical profile [38]. Bryostatin 1 is also known as NSC 339555, and its IUPAC name is described as [(1*S*,3*S*,5*Z*,7*R*,8*E*,11*S*,12*S*,13*E*,15*S*,17*R*,21*R*,23*R*,25*S*)-25-acetyloxy-1,11,21-trihydroxy-17-[(1*R*)-1-hydroxyethyl]-5,13-bis(2-methoxy-2-oxoethylidene)-10,10,26,26-tetramethyl-19-oxo-18,27,28,29-tetraoxatetracyclo [21.3.1.13,7.111,15]nonacos-8-en-12-yl](2*E*,4*E*)-octa-2,4-dienoate. Other identifications include CAS, PubChem and DrugBank IDs, i.e., 83314-01-6, 5280757 and DB 11752, respectively [16].

Regarding physical and chemical characteristics, this compound resembles a white solid [16], represented by the formula C_47_H_68_O_17_, with a molecular weight of 905 g/mol and exact mass of 904.445 Da. Bryostatin 1 can donate four hydrogen bonds and accept 17 hydrogen bonds [46]. With a Topological Polar Surface Area of 240 Å^2^, bryostatin shows high solubility in solvents such as *tert*-butanol, DMSO and ethanol; however, it can be classified as insoluble in water, with a solubility value of 0.00595 mg/mL [17,47]. In terms of stability, in its pure form, bryostatin 1 should be stored at −20 °C [17]. However, if dissolved in PET solvent and t-butanol, this compound can be stored for 24 and 48 h at ambient temperature or even 23 h in 50 °C, without degradation [16]. As stated in the National Library of Medicine (NIH), bryostatin 1 is considered an “antineoplastic agent” according to the MeSH Pharmacological Classification [48], although it has been used in clinical trials for various pathologies and conditions such as Alzheimer’s disease (AD), HIV infection, pain and fatigue [16]. However, in this article, we will only focus on its potential as an anticancer drug.

## 3. Chemistry and Synthesis of Bryostatin 1

**Figure 1:**
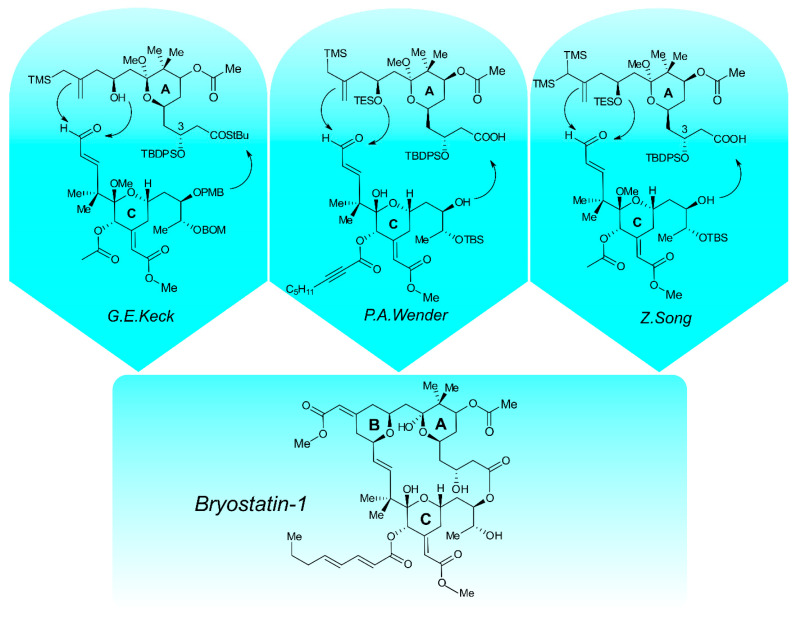
The A-ring and C-ring units described by Keck, Wender and Song as precursors for the synthesis of bryostatin 1. TMS—trimethylsilyl; PMB—*para*-methoxybenzyl; BOM—benzyloxymethyl; TES—triethylsilyl; TBDPS—*tert*-butyldiphenylsilyl; TBS—*tert*-butyldimethylsilyl.

Bryostatins are natural marine products with a promising biological activity. Over four decades of research, a family of 21 bryostatins has been identified [49]. Bryostatin 1 was originally isolated from the bryozoan *Bugula neritina* by Pettit and co-workers [50]. It is a highly oxygenated macrolide lactone with eleven stereocenters and incorporates three hydropyran rings. It is also the most commonly used variant in clinical trials. It was established that the true source of the bryostatin is not *B. neritina* but rather a bacterial symbiont “*Candidatus Endobugula sertula*” [51,52]. The meager quantity in marine biosome has severely limited further research and clinical applications. The eighteen grams of bryostatin 1 obtained from fourteen tons of *B. neritina* (0.00014% yield) by Pettit and co-workers demonstrate the scale of the limitations in the isolation of this compound [53]. Supercritical CO_2_ extraction improves the yield of the process [54]; however, sustainable harvesting of marine ecosystems and aquaculture of *B. neritina* is limited. To overcome supply limitations and facilitate the production of diverse analogues, attempts have been made to develop a total synthesis of bryostatin 1. Generally, the total synthesis of bryostatin 1 is generally divided into synthesis of functionalized A and C pyran rings, and “pyran annulation” leads to the formation of the B ring (Figure 1).

The first total synthesis of bryostatin 1 was reported by Keck and co-workers in 2011 [55]. The described method required 57 steps of the synthesis. The C-ring aldehyde was synthesized from (*E*)-5-hydroxy-2,2-dimethylpent-3-enoic acid protected by *tert*-butyldimethylsilyl group (TBS) esterified with homoallylic alcohol previously prepared from commercially available (*R*)-isobutyl lactate, followed by a Rainier metathesis reaction to obtain a glycal, converted in six steps to the southern fragment of bryostatin 1 [56]. Beginning from the previously reported allylstannane aldehyde, the next three steps led to the A-ring hydroxyallylsilane. The convergent union of the C-ring aldehyde with the A-ring hydroxyallylsilane resulted in the B-ring. It was discovered that the C3 hydroxyl group must be freed to seco acid before C1 hydrolysis, which is necessary for Yamaguchi macrolactonization to follow. After the union of the two above-mentioned fragments, the next eleven steps resulted in the final product [57].

In 2017, Wender reported that the total synthesis of bryostatin 1 proceeded in 29 stages, with a linear sequence of 19 steps and 4.8% overall yield. The synthesis of the C-ring subunit was started from dihydropyran. To reduce time, cost, and waste-generating chromatographies, many steps were conducted without product purifications. The overall synthetic route to the C-ring subunit proceeded in 13 steps, with an overall yield of 16%. The synthesis of the A-ring subunit began with the Claisen reaction between ethyl 3,3-diethoxypropanoate and *tert*-butyl acetate. The authors reported that the Evans–Saksena diastereoselective reduction of hydroxyketone, leading to cyclization and formation of a pyran ring performed in AcOH/MeCN, resulted in low diastereoselectivity (2:1), while the addition of acetone as a co-solvent improved diastereomeric ratio to over 15:1. The ten steps of the synthetic route completed the A-ring subunit in 13% overall yield. Both precursors were then coupled via Yamaguchi esterification and Prins macrocyclization to obtain bryostatin 1 in four sequential steps [58].

The total synthesis of the A-ring described by Song began from commercially available aldehyde: (*R*)-ethyl 3-hydroxy-5-oxopentanoate. During exploration of catalysts, *O*-benzyl-protected quinidine was identified as an agent allowing enantioselective control asymmetric acid chloride–aldehyde cyclocondensation at C5 with a good diastereomeric ratio (20:1) [59]. The final nickel-catalyzed Kumada cross-coupling yields the A-ring fragment in 90% yield. The proposed synthetic route of the northern fragment was completed in 11 linear steps and significantly reduced the number of purification steps (six purification steps), enhancing the overall synthetic efficiency (14.3% yield from (*R*)-ethyl 3-hydroxy-5-oxopentanoate). Song synthesized the southern fragment containing the C-ring starting from the commercially available (*R*)-1-chlorohex-5-en-2-ol. The optimization of palladium-catalyzed site-controlled isomerization of terminal olefin of starting alkene indicated 2-fluoroethanol as the most effective additive, with the *E*/*Z* isomer ratio of 15:1 [60]. As previously reported, though impractical for scale-up, Zn/Cu-promoted cross-coupling of enone and α-hydroxy iodide was resolved. Also, 7-((4-methoxybenzyl)oxy)-4,4-dimethylhepta-1,5-dien-3-one and (*E*)-triethyl((1-iodohex-4-en-2-yl)oxy) silane were efficiently coupled in the presence of tris(bipyridine)ruthenium(II) chloride (5 mol%), 440 nm blue LED irradiation, and continuous flow conditions coupling in 78% yield. The subsequent synthesis steps were performed with high yields, and the whole synthetic route of the southern fragment was completed in 12 linear steps with 8 purification steps and an overall yield of 18.5%. After combining the two above-mentioned fragments, an additional eight steps resulted in achieving the final product [61].

## 4. Mechanism of Action

### 4.1. Modulation of Protein Kinase C (PKC)

Bryostatin is a potent modulator of protein kinase C (PKC), interacting with this family of enzymes in a unique and complex way. The PKC family consists of a group of evolutionarily conserved serine/threonine kinases found across many species. Extensive research has explored the role played by PKC in both physiological processes and various diseases, demonstrating its participation in numerous metabolic functions in nearly all types of cells. PKC enzymes perform major functions in several signal transduction pathways that translate environmental signals into cellular responses, thereby influencing gene expression, cell cycle progression, migration, proliferation, differentiation, survival, and apoptosis [62,63].

Eleven PKC isoforms have been discovered and divided into three main groups. The classical (or conventional) PKCs, comprising α, βI, βII, and γ, require phosphatidylserine (PS), diacylglycerol (DAG), and Ca^2+^ for activation. In contrast, the novel PKCs (δ, ε, η, θ) depend on PS and DAG, while the atypical PKCs (ζ, ι/λ) are activated exclusively through protein–protein interactions, with human aPKCι corresponding to aPKCλ in mice [64]. The specific functions of individual PKC isoforms are determined by their receptor-mediated relocation to distinct intracellular locations. Disruptions or abnormalities in PKC activity have been linked to a range of diseases, including diabetes, heart failure, Alzheimer’s and Parkinson’s diseases, allergies, inflammatory conditions, and various autoimmune disorders [65,66]. Moreover, PKC has been associated with several types of cancer affecting different organs, which suggests that these isoforms might serve as valuable targets for therapeutic interventions in various human diseases [67,68]. For instance, PKCα is broadly found in cardiac, neural, liver, and immune cells, where it regulates processes like proliferation, differentiation, survival, and apoptosis. This enzyme plays a crucial role in maintaining proper heart function and neural communication, and its dysregulation is associated with various cancers, heart diseases and other disorders [64,69]. In turn, PKCβ plays a key role primarily in B cells, macrophages and heart tissue, where it influences immune function, cell growth and glucose regulation. Dysregulated expression of PKCβ is often linked to the development of diabetes and certain cancers, including B-lymphoblastic lymphoma and acute myeloid leukemia [64]. In contrast, PKCγ is highly expressed in the brain, especially within the cerebellum, where it is involved in synaptic plasticity as well as learning and memory functions [70].

Bryostatin 1 exerts diverse biological effects by interacting with PKC. Upon binding to the *N*-terminal C1 domain of PKC, its ring structures adopt a cap-like configuration. Although bryostatin 1 is a hydrophilic compound, it binds to PKC with high affinity, thus demonstrating potency comparable to that of phorbol esters, which are typically hydrophobic ligands of PKC [71,72]. While bryostatin 1 is capable of interacting with various PKC isoforms, its binding affinity differs among them. Specifically, it binds to PKCα, PKCβ2, PKCδ and PKCε with affinities of 1.35, 0.42, 0.26, and 0.24 nM, respectively [40]. In vitro studies conducted in neuronal cell cultures showed that bryostatin 1 strongly activates PKCα, PKCδ and PKCε at concentrations of 10^−8^, 10^−9^ and 10^−10^ M, respectively. Time-course studies revealed that 10^−10^ M bryostatin 1 significantly activated PKCε within 30 min and PKCδ after 1 h [73]. These findings suggest that bryostatin 1 exhibits a greater selectivity for PKCε and PKCδ, with a particular preference for PKCε. This isoform-specific affinity was also evident in in vivo experiments [74]. Upon binding, bryostatin 1 rapidly promotes PKC activation by inducing its translocation to the membrane and triggering autophosphorylation [71].

The typical progression of PKC activity includes three distinct phases, namely an initial activation phase lasting less than 40 min, a prolonged downregulation phase spanning several hours, and a later phase of new enzyme synthesis occurring after more than 2 days [75]. PKC activation is driven by the enzyme’s translocation from the cytoplasm to the cell membrane in response to second messengers. This is followed by enzyme downregulation, which results from its ubiquitination and degradation via the proteasome. Eventually, the breakdown products are recycled, leading to the de novo synthesis of PKC [76].

It is important to note that brief exposure to bryostatin leads to PKC activation, while prolonged treatment has been shown to reduce cellular PKC levels and functional inhibition, probably through ubiquitin-dependent proteasomal degradation. However, findings have been inconsistent, and certain bryostatin-induced effects may vary depending on the specific cell line used [77]. Bryostatin has demonstrated anticancer effects in vitro against various human leukemia cell lines, showing notable potency against B-cell-derived lymphoid malignancies, such as the WSU-DLCL2 large cell lymphoma. Conversely, in some cell lines, bryostatin has been linked to antiapoptotic outcomes, which are considered to result from its ability to induce serine phosphorylation of Bcl-2 and Bad proteins [78]. In another study conducted in the REH cell line, Ruvolo et al. [79] observed that bryostatin promoted the translocation of PKCα to the mitochondria, leading to Bcl-2 phosphorylation and enhanced resistance to chemotherapy.

Apart from its role in promoting cell survival, PKC also activates the extracellular signal-regulated kinase (ERK) signaling pathway, which is essential for bryostatin-induced differentiation of leukemia cells. This includes B-lymphoid differentiation in REH acute lymphoblastic leukemia cells. In B-cell chronic lymphocytic leukemia (B-CLL), bryostatin has been shown in vitro to promote differentiation toward a hairy cell leukemia (HCL)-like phenotype, marked by increased expression of CD11c, CD22, and tartrate-resistant acid phosphatase activity [80]. This transformation is believed to sensitize B-CLL cells to cytotoxic agents, particularly 2-chloro-2′-deoxyadenosine (2-Cda), an effective treatment for HCL. Supporting this, bryostatin enhanced the activity of 2-Cda in a drug-resistant B-CLL cell line (WSU-CLL) by altering the Bcl-2/Bax expression ratio, increasing Bax and decreasing Bcl-2 [81]. However, findings vary. Another study by Kitada et al. [82] reported increased Mcl-1 but no significant change in Bcl-2 or Bax after bryostatin treatment, and a reduction in both spontaneous and drug-induced apoptosis, which highlights cell line-dependent variability in response. Also, a study by Thomas et al. [77] revealed that bryostatin induces differentiation in B-CLL cells by triggering the ERK pathway in a PKC-dependent manner. Inhibition studies confirmed that the survival signals provided by bryostatin largely depend on PKC activation, indicating that bryostatin acts predominantly as a PKC agonist [77]. Although differentiation was achieved, the bryostatin-differentiated B-CLL cells became phenotypically distinct from HCL cells. Importantly, these cells display increased resistance to cell death induced by chemotherapeutic agents such as chlorambucil and purine nucleoside analogues. The authors found that the mechanism of action was not limited to differentiation; it also involved alterations in apoptosis-regulating protein levels, culminating in a broader resistance profile against cytotoxic agents [77].

It is also important to note that bryostatin 1 modulates the function of various proteins that regulate the cell cycle, though its impact on apoptosis varies. It transiently induces the expression of p21, an intracellular inhibitor of cdk2, leading to the dephosphorylation and subsequent inactivation of cdk2, a process that correlates with the suppression of tumor cell proliferation [83]. Additionally, bryostatin 1 disrupts the p21 upregulation usually triggered by the potent PKC activator phorbol myristate acetate (PMA) [84]. For instance, in U937 cells, pretreatment with deoxycytidine prevents the bryostatin 1-induced increase in p21 and instead promotes apoptosis [85].

In conclusion, bryostatin 1 acts as a potent modulator of protein kinase C. It binds with high affinity to the *N*-terminal C1 domain of PKC isoforms, which induces a conformational change that promotes translocation and autophosphorylation of the enzyme. This activation is central to its biological effects, positioning bryostatin 1 as a prominent PKC agonist. Although bryostatin 1 interacts with various PKC isoforms, it has a preferential selectivity towards PKCε and PKCδ, with activation observable even at very low concentrations. The rapid activation is followed by a classical sequence of events, i.e., an initial activation phase, a prolonged downregulation phase (often due to ubiquitin-mediated proteasomal degradation) and, ultimately, new enzyme synthesis if exposure is sustained.

### 4.2. Induction of Apoptosis in Cancer Cells Through Activation of Signaling Pathways

Compounds of natural origin are an important source of potential anticancer drugs due to their ability to induce apoptosis through the activation of various signaling pathways. Among other things, they act by modulating the PI3K/Akt/mTOR, MAPK/ERK or caspase pathways, leading to an imbalance between pro- and antiapoptotic proteins, loss of mitochondrial integrity and activation of mechanisms responsible for programmed cell death. Because of this mechanism, compounds of natural origin are gaining increasing interest as promising therapeutic agents for the treatment of various types of cancer [86,87,88]. Bryostatin 1, a macrocyclic lactone extracted from the marine invertebrate *Bugula neritina*, is known to be a potent modulator of protein kinase C (PKC) isoforms. This compound also transiently upregulates p21, a well-known inhibitor of cdk2, leading to the dephosphorylation and inactivation of cdk2, which correlates with a reduction in tumor cell proliferation. Simultaneously, bryostatin 1 can interfere with p21 induction by other PKC activators, and the resultant modulation of cell cycle regulators contributes to an altered apoptotic response that appears to be cell type-dependent [83]. The evidence suggests that while bryostatin’s mechanism as a PKC agonist is well defined, its overall impact on cancer cells is complex. It not only initiates processes like differentiation but may also confer resistance to chemotherapy by altering the balance of pro- and antiapoptotic proteins. These contrasting effects warrant a cautious approach to its clinical application. In cancer research, the compound has been shown to affect cell survival by modulating various signaling pathways leading to the induction of apoptosis. In U937 leukemia cells, bryostatin 1, in combination with cytarabine (ara-C), increased cytochrome c and Smac/DIABLO release, activation of caspases-3 and -9 and loss of the mitochondrial membrane potential, leading to apoptosis, even in cells overexpressing Bcl-xL. Similarly, in Reh leukemia cells, bryostatin 1 induced the ubiquitination and proteasomal degradation of Bcl-2, reducing its protein and mRNA levels, which suggests involvement of the ubiquitin-proteasome pathway in the regulation of antiapoptotic gene expression [89,90]. In contrast, in U937 leukemia cells, bryostatin 1, in combination with the proteasome inhibitor lactacystin, disrupted the PKC/MAPK pathway, leading to prolonged ERK phosphorylation and increased apoptosis. MEK inhibitors, such as PD98059, counteracted ERK activation and protected cells from death. This finding suggests a key role in the induction of apoptosis for this pathway [91].

In other studies, it has been shown that in U937 leukemia cells overexpressing Bcl-xL, bryostatin 1 enhanced paclitaxel-induced apoptosis by increasing mitochondrial damage and caspase activation, despite the presence of Bcl-xL. The effects of bryostatin 1 were mimicked by the MEK1/MAPK inhibitor PD98059, suggesting that modulation of the MAPK pathway plays an important role in this process [92]. In LNCaP prostate cancer cells, bryostatin 1 inhibited PKCδ translocation to the cell membrane, preventing TNF-α release and apoptosis induction by PMA. This antiapoptotic effect of bryostatin 1 may be related to its ability to modulate PKCδ localization and cytokine secretion [93]. In Toledo B-NHL lymphoma cells, bryostatin 1 activated the RasGRP-Ras-Raf-MEK-ERK pathway, leading to the phosphorylation of the proapoptotic protein Bim. Phosphorylation of Bim intensified its proapoptotic activity, resulting in Bax/Bak activation-dependent apoptosis, and increased mitochondrial membrane permeability [94].

**Figure 2:**
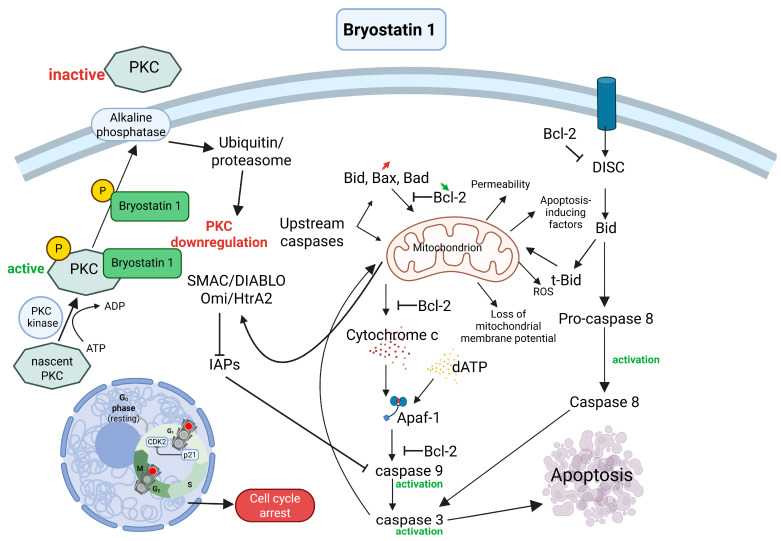
Potential mechanisms of action related to bryostatin 1. Abbreviations: PKC—protein kinase C, SMAC—second mitochondria-derived activator of caspase, DIABLO—direct IAP binding protein with low pI, Omi/HtrA2—proapoptotic mitochondrial serine protease, IAPs—inhibitors of apoptosis proteins, APAF—apoptotic protease activating factor, DISC—death-inducing signaling complex. The red and green arrows indicate increased and decreased expression, respectively.

The studies presented above only confirm the broad spectrum of action of bryostatin 1 involving activation of different signaling pathways in different tumor types, which demonstrates its antitumor properties (Figure 2).

### 4.3. Pharmacokinetics of Bryostatin 1

High-molecular-weight compounds, such as complex macrolides, inherently pose significant challenges in terms of bioavailability (particularly oral) and accurate assessment of their biodistribution. These difficulties often arise from limited gastrointestinal absorption, extensive first-pass metabolism, and strong binding affinity to tissue and plasma proteins, all of which frequently preclude reliable pharmacokinetic evaluation. This applies also to bryostatin 1, a marine-derived macrolide which, despite exhibiting potent biological activity at nanomolar concentrations, is primarily administered intravenously due to its extremely low oral bioavailability.

There is very little data available in the literature on the pharmacokinetic profile of bryostatin 1. A breakthrough in this area was the study by Zhang et al., conducted using a mouse model and a radioactively labeled form of [^3^H]-bryostatin 1. This approach allowed for precise monitoring of the distribution, metabolism, and elimination of the compound in vivo. It also demonstrated that most of the detected radioactivity was associated with the unchanged form of bryostatin 1, which indicates its high metabolic stability. The compound was widely distributed in tissues following intravenous (i.v.) administration, with the highest concentrations found in the lungs, liver, gastrointestinal tract, and adipose tissue, while its plasma levels declined rapidly. Moreover, the presence of bryostatin 1 was also detected in the brain, which confirms its ability to cross the blood–brain barrier. Although the compound’s presence in the central nervous system was short-lived, the measured concentrations were sufficient to activate PKC. Bryostatin 1 remained in the body in its unchanged form for at least 24 h after administration, further supporting its metabolic stability. During the first 12 h after i.v. administration, it was eliminated primarily with urine (23.0 ± 1.9% of the dose). By 72 h post-administration, radioactivity was excreted evenly through both the kidneys and the gastrointestinal tract (approximately 40% via each route).

The data reported by Zhang et al. serve as a reference point for evaluating the bioavailability of bryostatin 1. Their study showed that the compound followed a two-compartment pharmacokinetic model after intravenous administration, whereas a one-compartment model with first-order absorption was applied following intraperitoneal (i.p.) administration. The pharmacokinetic parameters determined in this study are summarized in Table 1. The use of an isotopically labeled compound allowed for not only precise quantification of tissue concentrations and distribution but also revealed a marked difference in systemic availability between i.v. and i.p. routes, favoring the latter when comparing AUC values [95].

However, determining bryostatin 1 concentrations in biological material from humans remains a significant challenge. Considering its low bioavailability after oral administration, in clinical studies on this compound, it is practically administered intravenously only. However, studies that applied a highly sensitive HPLC method using the SPE extraction technique detected concentrations of 12 ng/mL [96]. Despite this fact, in phase I clinical studies, no drug was detected in plasma samples taken from patients treated with bryostatin 1. Patients received various doses of bryostatin 1, ranging from 20 to 65 µg/m^2^ in a bolus or in a 24 h infusion at a dose of 50 µg/m^2^, with blood being collected in most cases at 1-hour intervals over a period of a half to 6 h. The fact that the compound was not detected in human biological material is most likely due to the strong lipophilicity and rapid distribution of the compound into tissues, which resulted in a rapid decrease in plasma concentration below the detection threshold of the method [96]. For many years, lack of appropriate analytical tools prevented full pharmacokinetic studies in humans. Subsequent approaches were based on the use of mass spectrometry coupled with liquid chromatography (LC-MS/MS), which allowed for determination of bryostatin 1 in human plasma with a higher precision, although still with limited effectiveness in clinical conditions. The developed analytical method was successfully used in clinical practice to determine bryostatin 1 concentration in plasma samples taken from a patient with cancer who was administered intravenous infusion of the drug at a dose of 20 μg/m^2^. During the infusion, on days 8 and 15 of the therapy, bryostatin 1 concentrations in plasma were in the range of 80–100 pg/mL. The half-life was estimated to be approximately nine hours, and the presence of the drug in the blood was confirmed four hours after the end of the infusion [97].

To compare, in the case of other macrolides with a similar structure and high molecular weight, such as rapamycin (sirolimus) or tacrolimus, it is also quite difficult to determine their plasma concentrations. It results from their high lipophilicity, strong binding to plasma proteins and extensive tissue distribution. For these compounds, appropriately sensitive LC-MS/MS methods have been developed with picomolar concentration determination [98]. However, bryostatin 1 seems to be characterized by an even lower bioavailability and a shorter detection period in the circulation, which makes it exceptionally difficult to quantitatively assess in patients, despite the use of advanced analytical methods. Nevertheless, animal studies have shown that bryostatin 1, as a natural macrocyclic lactone with a strong modulator effect on protein kinase C (PKC) isoforms, is active even at subnanomolar concentrations, and the fact that it penetrates the blood–brain barrier gives grounds for considering bryostatin 1 as a potential therapeutic agent not only against cancer but also neurodegenerative diseases.

## 5. Preclinical and Clinical Studies

### 5.1. In Vitro Studies of Bryostatin 1

In vitro studies in a cellular model are a key component of modern cell biology and biotechnology. They provide precision when identifying the molecular mechanisms that govern cellular function. These studies can reproduce near-physiological conditions, making it possible to analyze how cells respond to a variety of agents, such as drugs, toxins or environmental changes. Cellular models provide the basis for testing new therapies and also allow a better understanding of the processes involved in the development of diseases such as cancer, neurodegeneration or viral infections. This section focuses on the effects of bryostatin 1 in an in vitro model.

McGown et al. showed that the combination of bryostatin 1 with the anti-estrogen tamoxifen resulted in a significant synergistic increase in the growth inhibition of P388 (murine lymphocytic leukemia) cells in an in vitro model. In the present study, the inhibitory effect of bryostatin 1 in the presence of non-inhibitory concentrations of tamoxifen was increased approximately 200-fold, whereas the growth inhibition by tamoxifen in the presence of non-inhibitory concentrations of bryostatin 1 was increased over 30-fold [99].

In another study, Biberacher et al. showed that bryostatin 1 sensitizes chronic lymphocytic leukemia cells to the cytotoxic effects of BL22 via activation of protein kinase C and subsequent increased surface expression of CD22. Analysis revealed that protein kinase C activation additionally activates an autocrine feedback loop that degrades protein kinase C-βII. The authors demonstrated that, apart from primary chronic lymphocytic leukemia cells, bryostatin 1 sensitizes diffuse large B-cell lymphoma and mantle cell lymphoma cells to BL22-induced apoptosis [100]. Other examples of research are presented in Table 2 below.

### 5.2. In Vivo Studies on Bryostatin 1

In vivo studies play a key role in biological, medical and pharmaceutical research as they allow the assessment of biological processes throughout the body under natural physiological conditions. Unlike in vitro research, which is limited to analysis at the cellular or tissue level, in vivo research allows the effects of a compound, drug or therapy to be monitored in the context of the whole organism. The use of animal experimental models can provide valuable information on the pharmacokinetics, toxicity and potential efficacy of new therapies. This chapter outlines the different in vivo models used in biomedical research for bryostatin 1.

Koutcher et al. conducted an in vivo study to evaluate the effect of sequential administration of bryostatin 1 and paclitaxel in a mouse tumor model and correlated this effect with cell cycle events, tumor metabolism and tumor blood flow. Using phosphorus nuclear magnetic resonance (31P-NMR) spectroscopy in vivo, the authors showed that bryostatin 1 at a dose of 80 mg/kg induced a decrease in both intra-tumor pH and high-energy phosphates. In contrast, perfusion studies using dynamically enhanced NMR imaging with gadolinium diethylenetriamine pentaacetic acid also showed reduced tumor blood flow, while indicating that the inhibition of the tumor response to paclitaxel by bryostatin1 is multifactorial and involves factors as diverse as inhibition of cell entry into mitosis, reduction in pH and energy metabolism and, ultimately, reduced tumor blood flow [101].

In contrast, Zhang et al. investigated the preclinical pharmacology of bryostatin 1 in mice using [C26-3H]-labeled bryostatin 1 after i.v. and i.p. administration. It was shown that after i.v. administration, the plasma disappearance curve of bryostatin 1 was described by a two-compartment model, with half-lives of 1.05 and 22.97 h, respectively, and after single-compartment i.p. administration of 0.81 and 28.76 h, respectively. Additionally, the authors showed that bryostatin 1 was widely concentrated in the lungs, liver, gastrointestinal tract and adipose tissue, and that its concentration in the gastrointestinal tract, along with fecal excretion, suggests the possibility of an enterohepatic circulation. To sum up, these studies have shown that bryostatin 1 is relatively stable in vivo, widely distributed but concentrated in some major tissues and rapidly excreted first in urine and then in feces [95]. Other studies on the in vivo effect of bryostatin 1 are shown in Table 3 below.

### 5.3. Clinical Trials on Bryostatin 1

Clinical trials are the cornerstone of modern medicine, providing assessment of safety, efficacy and potential benefits of new therapies, drugs and medical procedures in real-world settings. To accurately determine the impact of new treatments on human health, the process involves close monitoring of patients at various stages, from preliminary trials to large-scale population studies. Clinical trials not only contribute to the development of innovative therapies but also form the basis for approval of medicines by relevant regulatory authorities. This chapter outlines the importance of clinical trials in the context of bryostatin 1.

In their study, Dowlati et al. showed that the maximum tolerated dose of bryostatin 1 in phase II was 50 g/m^2^/24 h, and vincristine 1.4 mg/m^2^ repeated every two weeks showed significant antitumor activity in a population of patients with relapsed disease, including patients in whom high-dose chemotherapy had failed. The clinical trial included five durable complete and partial responses and five patients with stable disease lasting more than 6 months (range between 6 and 48 months). All five patients who had an overall increase in CD5 cell apoptosis achieved a clinical response or prolonged stable disease. In this group, four individuals did not show an initial decrease in apoptosis at 6 h. Finally, the authors determined the recommended dose of bryostatin 1 in phase II to be a 24 h infusion followed by vincristine, repeated every 14 days. Myelotoxicity was minimal and muscle pain was dose-dependent [118].

In turn, Roberts et al. revealed that bryostatin 1 was safe and well-tolerated in combination with full-dose fludarabine (25 mg/m^2^/d × 5). The recommended dose of bryostatin 1 in phase II was 50 mg/m2 for both sequences, i.e., bryostatin 1—fludarabine and fludarabine—bryostatin 1. This combination was active against both chronic lymphocytic leukemia (CLL) and indolent lymphomas, and responses were observed in patients previously treated with fludarabine. However, correlative studies did not support the hypothesis that bryostatin 1 enhances the activity of fludarabine by reducing protein kinase C expression in target cells [119]. Additional clinical trials involving bryostatin 1 are presented in Table 4 below. 

## 6. Conclusions, Future Perspectives and Challenges

Bryostatin 1, a macrocyclic lactone of natural origin, exhibits multi-directional anticancer properties, mainly through selective modulation of protein kinase C (PKC). Its ability to affect different signaling pathways leads to the activation of apoptosis in different types of cancer cells. Also, bryostatin 1 regulates the expression of genes associated with cell survival and death, including increasing the levels of Bax, cytochrome c, Smac/DIABLO and decreasing the expression of Bcl-2. This leads to mitochondrial activation, disruption of the membrane potential and activation of caspases, eventually resulting in controlled cell death. Bryostatin 1 acts as a potent modulator and agonist of protein kinase C (PKC), selectively binding to the *N*-terminal C1 domain, particularly affecting PKCε and PKCδ isoforms. This interaction triggers enzyme activation, translocation, autophosphorylation, and subsequent downregulation. Additionally, bryostatin 1 transiently increases p21 expression, inhibiting cdk2 and reducing tumor proliferation, though its influence on cell cycle regulators and apoptosis is complex and varies by cell type. These contrasting effects, including potential chemotherapy resistance, indicate it should be applied with caution in clinical settings. Clinical trials to date have shown limited efficacy of bryostatin 1 as monotherapy; however, its synergism with classical chemotherapeutic agents, such as paclitaxel or cisplatin, suggests potential in combination therapy, particularly in refractory cancers. However, the low bioavailability, short half-life and systemic toxicity of bryostatin 1 remain significant challenges. In this context, nanotechnology offers new opportunities to improve the therapeutic efficacy of this compound. The development of targeted delivery systems for bryostatin 1, with the use of lipid nanoparticles, polymeric nanoparticles or micelles, can significantly increase its stability, reduce toxicity and enable selective accumulation in tumor tissue. For example, nanocarriers modified with ligands that recognize receptors overexpressed in tumor cells allow targeted delivery of the drug, thus increasing its efficacy while reducing side effects among patients. Biotechnological work is also underway to biosynthesize bryostatin 1 using modified microorganisms and to design synthetic analogues with a more favorable pharmacological profile. Combined with nanotechnology and immunotherapy, bryostatin 1 could form part of future targeted therapies, particularly for cancers that are difficult to treat with conventional methods. The challenges largely stem from its complex, context-dependent biological effects, particularly through PKC modulation, cell type-specific responses, and inconsistent pro- versus antiapoptotic outcomes.

In conclusion, although further research is required, the potential of bryostatin 1 to activate apoptosis through multiple signaling pathways, its effect on the expression of genes regulating cell survival and its potential for synergy with modern technologies, such as nanocarriers and genetic engineering, make it a promising candidate in the development of innovative cancer therapies.

## Figures and Tables

**Table 1:** Selected pharmacokinetic parameters of bryostatin 1 in mice depending on the route of administration [95].

Parameter	Intravenous (i.v.)	Intraperitoneal (i.p.)
Pharmacokinetic model	Two-compartment	One-compartment with first-order absorption
T_1_/_2_ (distribution)	1.05 h	–
T_1_/_2_ (elimination)	22.97 h	28.76 h
T_1_/_2_ (absorption)	–	0.81 h
AUC	376.7 ng/mL * h	620.2 ng/mL * h
Cmax	92.9 ng/mL	13.5 ng/mL
Volume of distribution	2.37 mL/kg	2.72 mL/kg
Clearance	0.11 mL/(kg * h)	0.06 (kg * h)
Elimination route	Urine + feces	Urine + feces
Brain penetration	Rapid, short-lasting	Longer duration, lower intensity

**Table 2:** In vitro studies of anticancer activity of bryostatin.

Cell Line	IC_50_	Activity/Mechanism/Effects	Ref.
MKN-74 gastric cancer cell line.	1 µM	Treatment of tumor cells with bryostatin 1 before paclitaxel decreases mitotic entry of the cells.	[101]
WSU-CLL human chronic lymphocytic leukemia cell line.	Bryostatin 1: 200 nM Auristatin-PE: various Dolastatin 10: various	There is a synergetic effect between these agents (auristatin-PE, dolastatin 10) and bryostatin 1 as regards apoptosis and cell death. It is more apparent in the bryostatin 1 + auristatin PE combination.	[102]
Human cervical carcinoma HeLa cells. HeLa cisplatin-resistant variants (HeLa/CP).	1 nmol/L	PKCy acts as a proapoptotic protein but in full length may inhibit cisplatin-induced cell death. Persistent activation/downregulation of PKCy by bryostatin 1 leads to cisplatin sensitization. Pretreatment with bryostatin 1 enhanced cisplatin-induced cell death in HeLa and HeLa cells, overexpressing PKCy gene to 30% and 19%, respectively. Bryostatin 1 decreased the IC_50_ of cisplatin from 6.4 to 1.7 µmol/L in HeLa cells and >30 µmol/L to and 14 µmol/L in HeLa/CP cells.	[103]
NIH 3T3 fibroblast cell line.	Bryostatin 1: 0.01; 0.1; 1; 10; 100; 1000 nM Combination: 1 µM of both Bryostatin 1 and PMA	Bryostatin 1 was more potent than PMA in terms of translocating and downregulating PKCα, -δ, and -ε.	[104]
Dendritic cells (DC) derived from bone marrow of C75BL/6 female mice.	0.1; 0.5; 1; 5; 10; 50 nM	Bryostatin 1 induced CC chemokine release from bone marrow DC, including CCL2 and CCL3, via ERK pathway in a dose- and time-dependent way. Maximal effect observed at 5 and 10 nM. No significant induction occurred at 50 nM. Bryostatin 1 is a strong adjuvant which potentiates a peptide-based cancer vaccine.	[105]
B16 melanoma cells and REH human leukemia cells.	10^−10^; 10^−9^; 10^−8^; 10^−7^; 10^−6^ M	Bryostatin 1 has a direct antiproliferative effect against B16 melanoma, more potent as 10^−6^ M, but shows no evidence that it can stimulate nonspecific cell-mediated cytotoxicity. Against REH, bryostatin 1 showed a significant antiproliferative effect over all the concentrations.	[106]
WSU-DLCL2 human diffuse large cell lymphoma cells.	10 nM	Bryostatin 1 increased Bax expression but only modestly induced apoptosis. Adding bryostatin 1 to CHO (cyclophosphamide monophosphate) leads to an inhibition of the cell growth by over 100%.	[107]
4TBM and 4THM cells originated from 4 T1 breast cancer cells.	10 ng/mL	The inhibitory effects of bryostatin 1 on chemokine secretion induced by CXCR2 antagonist seems to be mediated mainly by PKCδ followed by PKCε.	[108]
Leukemia cells from heparinized peripheral blood of patients with B-CLL.	10^−6^ to 10^−10^ mol/L	Bryostatin 1 can induce differentiation of B-CLL cells.	[109]
Human leukemia cell lines: HL-60 and K562. T lymphoblast cells: CEM. REH Human Lymphoblastic leukemia cells.	10^−11^ to 10^−7^ mol/L	Bryostatin 1 (possibly by activation of protein kinase C) inhibits clonogenic leukemia cells at concentrations that stimulate normal hematopoietic progenitors. Bryostatin 1 may be valuable in the treatment of leukemias and MDS.	[110]
Peripheral Blood Mononuclear Cells (PBMCs).	1 pM–100 nM	Bryostatin 1 increased IL-2 receptor expression on CD4+, CD8+ and CD56+ cells. Maximal IL-2 receptor induction and proliferation at 10 nM.	[111]
HEK293 Human Embryonic Kidney and Murine Bone Marrow-derived dendritic cells (DCs).	10 ng/mL	Bryostatin 1-induced production of IFN-β, MIP1-α, and RANTES, regulated, at least in part, through TLR4 activation. Also induced NF-kB activation, with TLR4 involved. TLRs are involved in Bryostatin 1-mediated activation of IRF-3, specifically via TLR4 in BMDCs.	[112]
Bone Marrow Dendritic Cells generated from C57BL/6 mice.	10 nM	Bryostatin 1 caused the maturation of DCs with a significant increase in the expression of CD40, CD80 and CD86. Bryostatin 1-induced activation of dendritic cells is not mediated by low levels of endotoxin contamination.	[112]
KB-3-1 Human papillomavirus related cervical adenocarcinoma cells. KB-C1 carcinoma cells. HeLa-MDR1-G185 multidrug-resistant HeLa cells transfected with a wild-type multidrug resistance gene 1 (MDR1). HeLa-MDR1-V185 multidrug-resistant HeLa cells transfected with a mutant MDR1 gene.	1 µM	Bryostatin 1 is able to reverse resistance to vinblastine, colchicine and adriamycin in cells V185, but not in the parental cells expressing G185. In cells expressing MDR1-V185, bryostatin 1 can inhibit the efflux of the PGP-substrate rhodamine 123 but not in the G185-expressing cells. Bryostatin 1 specifically reverses a mutant PGP.	[113]
WSU-DLCL2 human diffuse large cell lymphoma cells.	200 nM	Bryostatin 1 decreases P-glycoprotein by downregulating MDR1 expression. Exposure of culture cells to bryostatin 1 reversed the multidrug resistance phenotype within 24 h. A four-fold increase in vincristine accumulation was recorded in bryostatin l-treated cells compared with the control group.	[114]
LNCaP prostate cancer cells.	0.1; 1; 10; 100 nM	Bryostatin 1 is not an efficient killing agent for LNCaP prostate cancer cells. Moreover, as it inhibits the effect of other anticancer drugs, bryo-1 possesses antiapoptotic activity in LNCaP cells.	[93]
4T1 mouse breast cancer cells.	20; 200 and 400 nM	Approximately 60% of 4T1 cells undergo apoptosis within 48 h of treatment with bryostatin 1. Bryostatin 1-mediated effects on growth and apoptosis of 4T1 cells are not related to its ability to enhance translocation or downregulation of total PKC or PKC isoforms α and δ.	[115]
REH human acute lymphoblastic leukemia cells.	1 nM	Bryostatin 1 induces the ubiquitination, proteasome degradation and downregulation of the proto-oncogene Bcl-2 in human pre-B acute lymphoblastic leukemia cells. Bcl-2 acts like a multidrug resistance protein and its increased expression can protect tumor cells from the cytotoxic effects of most anticancer drugs. In contrast, decreased expression of Bcl-2 is associated with improved response to chemotherapy.	[90]

**Table 3:** In vivo studies on anticancer activity of bryostatin 1.

Organism	Dose	n	Exposure Time	Activity/Mechanism/Effects	Ref.
BALB/c nude mice	5, 25 and 50 µg/kg/day	15	n/a (i.p. injection)	Bryostatin 1 treatment hindered development of liver tumors as there were remissions in tumor size and invasion. This treatment had no adverse effects on the mice.	[116]
Four-week-old female ICR-SCID mice	75 µg/kg/ injection	20	n/a (s.c. injection)	Bryostatin is considered active in terms of tumor growth inhibition. Auristatin PE with bryostatin 1 is highly active in tumor growth inhibition and delay and kill value. In this group, all the mice treated were free of tumors for 150 days and thus were considered cured. The combination of dolastatin 10 with bryostatin 1 was considered active and only two of five mice were free of tumor. Bryostatin 1 in doses of 100 and 75 µg/kg were considered too toxic if administered alone and together with the other drugs, respectively.	[102]
C57BL/6 mice	Bryostatin 1: 20 ng E7 peptide: 20 µg	4–6	n/a (s.p. injection)	Bryostatin1 induces the CC chemokines release from BMDC, including CCL2 and CCL3. The Bryostatin 1 is a potent adjuvant which potentiates a peptide based cancer vaccine.	[105]
Female C57BL	1, 10, 50, 100 µg/kg as i.p. injections in 0.5 mL, during 5 days	6	n/a (i.p. injection)	Short term treatment of animals causes a dramatic reduction in the number of identified pulmonary melanoma metastases. Bryostatin 1 may be easily combined with other anticancer therapies without significant toxicity.	[106]
SCID mice	Bryostatin 1: 75 mg/kg, Cyclophosphamide: 40 mg/kg, Doxorubicin: 3.3 mg/kg, Vincristine: 0.5 mg/kg Prednisone: 0.2 mg/kg	39	n/a (i.p. injection)	The combination of Bryostatin 1 with CHOP (cyclophosphamide, doxorubicin, vincristine and prednisone) is highly active in tumor growth delay and inhibition, even better than Bryostatin 1 or CHOP alone.	[107]
Seven-week-old female BALB/c nude mice	Bryostatin 1: 5, 10, 15, 20, 25, 30 µM	48 la	n/a (not described)	Bryostatin 1 inhibited the development and progression of skin tumors in mice through the prevention of inflammation-inducing processes (such as activation of COX-2) and the quenching of radicals (H_2_O_2_). The 30 µM dose was the most effective in reducing inflammation and tumor progression.	[117]
Adult (from six to eight weeks old) female C57BL/6 mice	75 µg/kg body weight	Not described	n/a (i.v. injection)	Bryostatin 1 triggered a TLR-4-dependent T helper cell 2 (Th2) cytokine response, which can help treat disorders that are mediated by Th1inflammatory cells, and expanded a subset of myeloid dendritic cells that expressed a CD11c^+^ CD8^+^-CD11b^+^ CD4^+^ phenotype. Bryostatin 1 can act as a TLR4 ligand and activate innate immunity.	[112]
SCID mice	Bryostatin 1: 50–75 µg/kg Vincristine: 0.5 mg/kg Doxorubicin: 3.3 mg/kg Ara-C: 450 mg/kg	50	n/a (i.p. injection)	The combination of Bryostatin 1 and Vincristine is well tolerated and was associated with significant antitumor activity, regarding tumor growth delay and inhibition. Bryostatin 1 downregulates MDR1 in tumor tissue, enhancing significantly vincristine-induced tumor regression. Bryostatin 1 in combination with Doxorubicin or Ara-C was too toxic or had low antitumor activity, respectively.	[114]
From five- to six-week-old BALB/c mice	75 µg/kg body weight/injection	15	n/a (i.p. injection)	Bryostatin 1 inhibits both primary tumor growth and lung metastasis by 50%. It is equally effective in decreasing tumor if it is administered immediately after tumor challenge or several days after the detection of palpable tumors. Bryostatin 1 can effectively inhibit growth and metastasis of mammary tumor (especially p53 mutant or p53-null), although not eradicate tumors.	[115]

**Table 4:** Clinical trials conducted on anticancer activity of bryostatin 1. Information on dose, exposure time, schedule, number of patients and diseases they had, as well as descriptions of observations and conclusions on each trial.

Trial	Dose and Schedule	Disease	No. of Patients	Exposure Time	Activity/Mechanism/Effects	Ref.
Phase II	35–40 µg/m^2^ on days 1, 8 and 15 of each four-week cycle.	Renal cell carcinoma	32	1 h	Limited antitumor activity as a single agent. Common toxicities: myalgias, dyspnea, fatigue.	[120]
Phase I	5; 10; 20; 35; 50; 65 µg/m^2^.	Ovarian carcinoma, sarcoma, colon cancer, mesothelioma	19	1 h	No antitumor activity was observed. Recommended dose for phase II studies is 35–50 µg/m^2^ twice a week. The dose-limiting toxicity was myalgia.	[121]
Phase I	25 µg/m^2^ weekly; 35 µg/m^2^ biweekly; 25 µg/m^2^ for three weeks in a four-week cycle.	Non-small cell lung cancer, carcinoid tumor, esophageal carcinoma, small cell lung cancer, breast carcinoma, ovarian adenocarcinoma, hypernephroma, maxillary carcinoma, malignant melanoma, colorectal carcinoma, adenocarcinoma of unknown origin and pancreatic carcinoma	35	1 h	The dose-limiting toxicity was myalgia. Plasma IL-6 and TNF-α increased, and antitumor activity against malignant melanoma was observed early in the treatment.	[122]
Case Report	Bryostatin 1 (120 µg/m^2^) followed by 2-CdA (0.06 mg/kg) for five days and three cycles of chemotherapy.	Resistant chronic lymphocytic leukemia	1 (69-year-old male patient)	72 h	Decreased significantly peripheral blood lymphocytes (CLL cells), while simultaneously increasing their differentiation. Activated Bax/Bcl-2 apoptotic pathway, increasing its ratio. Enhanced sensitivity to 2-hCdA.	[123]
Phase II	Group 1—25 µg/m^2^ and escalated to 35 mg/m^2^. Group 2—25 µg/m^2^ to 35 µg/m^2^. Doses were given weekly followed by a week break, in four-week cycles.	Advanced colorectal cancer	28	24 h	Bryostatin 1 showed no complete or partial responses regarding antitumor activity. A different treatment schedule might enhance its effect.	[124]
Phase II	Group 1—25 µg/m^2^ weekly; Group 2—120 µg/m^2^ biweekly.	Metastatic melanoma	32	24 h (group 1) 72 h (group 2)	Although seven patients had stable disease, it was concluded that there were no partial or complete responses as the remaining patients had early progressive disease. The study treatment was not apparently effective.	[125]
Phase I	Doses were given every two weeks with escalation: 12; 20; 30; 42; 75; 120; 180 µg/m^2^.	Chronic lymphocytic leukemia Non-Hodgkin’s lymphoma	29	72 h	The maximum tolerated and recommended dose for phase II is 120 pg/m^2^ (40 pg/m^2^/d for three days). Myalgia was the dose-limiting toxicity.	[126]
Phase I	25; 35; 50 µg/m^2^ weekly for eight weeks	Advanced malignancy: ovarian carcinoma, renal carcinoma, melanoma, liposarcoma and low-grade non-Hodgkin’s lymphoma	19	24 h	Myalgia was the dose-limiting toxicity. The maximum tolerated and recommended dose for phase II trial was 25 µg/m^2^ per week. Partial and minor responses were seen both in four patients, two of them with ovarian carcinoma and the other two with low grade non-Hodgkin’s lymphoma.	[127]
Phase II	120 µg/m^2^ every two weeks. If disease progressed, patients could receive vincristine (with dose escalation) after completing bryostatin 1.	Non-Hodgkin’s lymphoma, Chronic lymphocytic leukemia	25	72 h	Bryostatin 1 alone resulted in one complete remission and two partial remissions. Sequential therapy with vincristine in doses up to 2 mg is feasible and well tolerated.	[128]
Phase I	Gemcitabine (mg/m^2^), followed by bryostatin 1 (µg/m^2^) on days 1, 8, and 15 of a 28-day cycle with dose escalation: 600/25; 800/25; 1000/25; 1000/30; 1000/35; 1000/45, respectively.	Nonhematologic cancer refractory to conventional treatment	36	24 h	Two heavily pretreated patients had a partial response, lasting 22 and eight months. Eight patients had stable disease. Three patients with non-small cell lung cancer had disease stabilization for more than four months. The combination of bryostatin 1 and gemcitabine is well-tolerated with limited grade 3 toxicity. The recommended dose of bryostatin 1 in combination with full doses of gemcitabine is 35 µg/m^2^.	[129]
Phase II	25 µg/m^2^ weekly for three weeks followed by a rest week.	Malignant melanoma	15	1 h	Toxicity in this study, apart from myalgia, was low. Bryostatin 1 is not effective as a single agent in this disease. Evidence suggests it may warrant further study in combination with cytotoxic or biological agents.	[130]
Phase I	Bryostatin 1: 12.5 escalated to 50 µg/m^2^ in increments of 12.5 µg/m^2^. HiDAC: 1.5 or 3 g/m^2^ dose every 12 h for two days	Refractory/relapsed acute leukemia: acute myelogenous leukemia, acute lymphoblastic leukemia, blast crisis in chronic myeloid leukemia	30	24 h	The combination of bryostatin 1 with HiDAC demonstrated some activity in heavily pretreated leukemia patients. Bryostatin 1 modulated PKC, induced apoptosis and showed potential synergy with HiDAC. The maximum tolerated dose was 50 µg/m^2^.	[131]
Phase Ib	25 µg/m^2^ weekly for three weeks or 12.5 µg/m^2^ on days 1 and 4 of each week.	Refractory malignancies: pancreatic cancer, breast cancer, melanoma, colon cancer, non-small cell lung cancer, soft tissue sarcoma, kidney cancer.	12	30 min (12.5 µg/m^2^); 1 or 24 h (25 µg/m^2^)	Three patients (with sarcoma, pancreatic and breast cancer), had stable disease for at least eight weeks, while eight patients showed disease progression. Lower bryostatin 1 doses may be more appropriate when this agent is intended as an immunomodulator since the split doses showed an increase in IL-2. PKC activity reductions were observed in some patients.	[132]
Phase II	Bryostatin 1: 45 μg/m^2^ followed immediately by Cisplatin: 50 mg/m^2^.	Recurrent and persistent epithelial ovarian cancer	8	72 h (Bryostatin 1) and 1 h (Cisplatin)	There was a modest response rate in patients with recurrent or persistent ovarian cancer treated with the combination of bryostatin and cisplatin. Its severe incidence of myalgias shown in this study limits its ability to be delivered in effective doses as patients required narcotics for pain control.	[133]

## Data Availability

Not applicable.

## References

[1] World Health Organization (WHO) Cancer. https://www.who.int/news-room/fact-sheets/detail/cancer.

[2] Cancer TODAY DataViz. https://gco.iarc.fr/today/en/dataviz/pie?mode=population&group_populations=0.

[3] Sharifi-Rad J., Ozleyen A., Boyunegmez Tumer T., Oluwaseun Adetunji C., El Omari N., Balahbib A., Taheri Y., Bouyahya A., Martorell M., Martins N. (2019). Natural Products and Synthetic Analogs as a Source of Antitumor Drugs. Biomolecules.

[4] Naeem A., Hu P., Yang M., Zhang J., Liu Y., Zhu W., Zheng Q. (2022). Natural Products as Anticancer Agents: Current Status and Future Perspectives. Molecules.

[5] Hashem S., Ali T.A., Akhtar S., Nisar S., Sageena G., Ali S., Al-Mannai S., Therachiyil L., Mir R., Elfaki I. (2022). Targeting Cancer Signaling Pathways by Natural Products: Exploring Promising Anti-Cancer Agents. Biomed. Pharmacother..

[6] Gupta A.P., Pandotra P., Sharma R., Kushwaha M., Gupta S. (2013). Marine Resource: A Promising Future for Anticancer Drugs. Stud. Nat. Prod. Chem..

[7] Khalifa S.A.M., Elias N., Farag M.A., Chen L., Saeed A., Hegazy M.-E.F., Moustafa M.S., Abd El-Wahed A., Al-Mousawi S.M., Musharraf S.G. (2019). Marine Natural Products: A Source of Novel Anticancer Drugs. Mar. Drugs.

[8] Tamzi N.N., Rahman M.M., Das S. (2024). Recent Advances in Marine-Derived Bioactives towards Cancer Therapy. Int. J. Transl. Med..

[9] Barreca M., Spanò V., Montalbano A., Cueto M., Díaz Marrero A.R., Deniz I., Erdoğan A., Lukić Bilela L., Moulin C., Taffin-de-Givenchy E. (2020). Marine Anticancer Agents: An Overview with a Particular Focus on Their Chemical Classes. Mar. Drugs.

[10] Wu L., Ye K., Jiang S., Zhou G. (2021). Marine Power on Cancer: Drugs, Lead Compounds, and Mechanisms. Mar. Drugs.

[11] Sun M.-K., Alkon D.L. (2006). Bryostatin-1: Pharmacology and Therapeutic Potential as a CNS Drug. CNS Drug Rev..

[12] Kortmansky J., Schwartz G.K. (2003). Bryostatin-1: A Novel PKC Inhibitor in Clinical Development. Cancer Investig..

[13] Trindade-Silva A.E., Lim-Fong G.E., Sharp K.H., Haygood M.G. (2010). Bryostatins: Biological Context and Biotechnological Prospects. Curr. Opin. Biotechnol..

[14] Smithsonian Environmental Research Center Bugula Neritina. https://invasions.si.edu/nemesis/species_summary/-95.

[15] Sharp K.H., Davidson S.K., Haygood M.G. (2007). Localization of “Candidatus Endobugula Sertula” and the Bryostatins throughout the Life Cycle of the Bryozoan Bugula Neritina. ISME J..

[16] National Library of Medicine (NIH) Bryostatin 1. https://pubchem.ncbi.nlm.nih.gov/compound/5280757.

[17] Sigma Aldrich NMR Chemical Shifts of Impurities. https://www.sigmaaldrich.com/MX/en/technical-documents/technical-article/genomics/cloning-and-expression/blue-white-screening.

[18] Saeed A.F.U.H., Su J., Ouyang S. (2021). Marine-Derived Drugs: Recent Advances in Cancer Therapy and Immune Signaling. Biomed. Pharmacother..

[19] Quitério E., Soares C., Ferraz R., Delerue-Matos C., Grosso C. (2021). Marine Health-Promoting Compounds: Recent Trends for Their Characterization and Human Applications. Foods.

[20] DrugBank Trabectedin. https://go.drugbank.com/drugs/DB05109.

[21] Smithsonian Environmental Research Center Ecteinascidia Turbinata. https://invasions.si.edu/nemesis/species_summary/159142.

[22] Larsen A.K., Galmarini C.M., D’Incalci M. (2015). Unique Features of Trabectedin Mechanism of Action. Cancer Chemother. Pharmacol..

[23] Gadducci A., Cosio S. (2022). Trabectedin and Lurbinectedin: Mechanisms of Action, Clinical Impact, and Future Perspectives in Uterine and Soft Tissue Sarcoma, Ovarian Carcinoma, and Endometrial Carcinoma. Front. Oncol..

[24] O’Shaughnessy J., Kaklamani V., Kalinsky K. (2019). Perspectives on the Mechanism of Action and Clinical Application of Eribulin for Metastatic Breast Cancer. Future Oncol..

[25] Shetty N., Gupta S. (2014). Eribulin Drug Review. South Asian J. Cancer.

[26] Yu M.J., Zheng W., Seletsky B.M., Littlefield B.A., Kishi Y. (2011). Case History: Discovery of Eribulin (HALAVEN^TM^), a Halichondrin B Analogue That Prolongs Overall Survival in Patients with Metastatic Breast Cancer. Annu. Rep. Med. Chem..

[27] Seshadri P., Deb B., Kumar P. (2021). Multifarious Targets beyond Microtubules—Role of Eribulin in Cancer Therapy. Front. Biosci.-Sch..

[28] Alonso-Álvarez S., Pardal E., Sánchez-Nieto D., Navarro M., Caballero M.D., Mateos M.V., Martin A. (2017). Plitidepsin: Design, Development, and Potential Place in Therapy. Drug Des. Dev. Ther..

[29] Washington Invasive Species Council Tunicate. https://invasivespecies.wa.gov/priorityspecies/tunicate/.

[30] Losada A., Muñoz-Alonso M.J., García C., Sánchez-Murcia P.A., Martínez-Leal J.F., Domínguez J.M., Lillo M.P., Gago F., Galmarini C.M. (2016). Translation Elongation Factor EEF1A2 Is a Novel Anticancer Target for the Marine Natural Product Plitidepsin. Sci. Rep..

[31] (2018). European Medicines Agency Annex Scientific Conclusions and Grounds for Refusal Presented by the European Medicines Agency.

[32] Nigam M., Suleria H.A.R., Farzaei M.H., Mishra A.P. (2019). Marine Anticancer Drugs and Their Relevant Targets: A Treasure from the Ocean. DARU J. Pharm. Sci..

[33] DrugBank Cytarabine. https://go.drugbank.com/drugs/DB00987.

[34] Cerrano C., Pansini M., Valisano L., Calcinai B., Sarà M., Bavestrello G. (2004). Lagoon sponges from carrie bow cay (Belize): Ecological benefits of selective sediment incorporation. BMIB-Boll. Dei Musei E Degli Ist. Biol..

[35] DrugBank Vidarabine. https://go.drugbank.com/drugs/DB00194.

[36] Sagar S., Kaur M., Minneman K.P. (2010). Antiviral Lead Compounds from Marine Sponges. Mar. Drugs.

[37] Shilpi J.A., Uddin S.J. (2020). Analgesic and Antipyretic Natural Products. Annu. Rep. Med. Chem..

[38] Montalvão S.I.G.H.M., Singh V., Haque S. (2014). Bioassays for Bioactivity Screening. Compr. Anal. Chem..

[39] DrugBank Ziconotide. https://go.drugbank.com/drugs/DB06283.

[40] Raghuvanshi R., Bharate S.B. (2020). Preclinical and Clinical Studies on Bryostatins, a Class of Marine-Derived Protein Kinase c Modulators: A Mini-Review. Curr. Top. Med. Chem..

[41] Davidson S.K., Haygood M.G. (1999). Identification of Sibling Species of the Bryozoan Bugula Neritina That Produce Different Anticancer Bryostatins and Harbor Distinct Strains of the Bacterial Symbiont “Candidatus Endobugula Sertula”. Biol. Bull..

[42] Slocum S.T., Lowell A.N., Tripathi A., Shende V.V., Smith J.L., Sherman D.H. (2018). Chemoenzymatic Dissection of Polyketide β-Branching in the Bryostatin Pathway. Methods Enzymol..

[43] Cragg G.M., Newman D.J. (2010). Nature as Source of Medicines; Novel Drugs from Nature; Screening for Antitumor Activity. Compr. Nat. Prod. II.

[44] Kedei N., Lewin N.E., Géczy T., Selezneva J., Braun D.C., Chen J., Herrmann M.A., Heldman M.R., Lim L., Mannan P. (2013). Biological Profile of the Less Lipophilic and Synthetically More Accessible Bryostatin 7 Closely Resembles that of Bryostatin 1. ACS Chem. Biol..

[45] Trost B.M., Wang Y., Buckl A.K., Huang Z., Nguyen M.H., Kuzmina O. (2020). Total Synthesis of Bryostatin 3. Science.

[46] Evans D.M., Carter P.H., Carreira E.M., Charette A.B., Prunet J.A., Lautens M. (1999). Total Synthesis of Bryostatin 2. J. Am. Chem. Soc..

[47] DrugBank Bryostatin 1: Uses, Interactions, Mechanism of Action|DrugBank Online. https://go.drugbank.com/drugs/DB11752.

[48] National Library of Medicine (NIH) Antineoplastic Agents—MeSH—NCBI. https://www.ncbi.nlm.nih.gov/mesh/68000970.

[49] Guo Q., Yang Y., Zhang H., Wang D., Li B., Jiang D., Tu X., Gao X., Zhang C., Qin Y. (2025). Total Syntheses of Bryostatins 1, 7, 9 and 9-N3. Angew. Chem. Int. Ed..

[50] Pettit G.R., Herald C.L., Doubek D.L., Herald D.L., Arnold E., Clardy J. (1982). Isolation and Structure of Bryostatin 1. J. Am. Chem. Soc..

[51] Sudek S., Lopanik N.B., Waggoner L.E., Hildebrand M., Anderson C., Liu H., Patel A., Sherman D.H., Haygood M.G. (2007). Identification of the Putative Bryostatin Polyketide Synthase Gene Cluster from “CandidatusEndobugula Sertula”, the Uncultivated Microbial Symbiont of the Marine BryozoanBugula Neritina. J. Nat. Prod..

[52] Miller I.J., Vanee N., Fong S.S., Lim-Fong G.E., Kwan J.C. (2016). Lack of Overt Genome Reduction in the Bryostatin-Producing Bryozoan Symbiont “Candidatus Endobugula Sertula”. Appl. Environ. Microbiol..

[53] Schaufelberger D.E., Koleck M.P., Beutler J.A., Vatakis A.M., Alvarado A.B., Andrews P., Marzo L.V., Muschik G.M., Roach J., Ross J.T. (1991). The Large-Scale Isolation of Bryostatin 1 from Bugula Neritina Following Current Good Manufacturing Practices. J. Nat. Prod..

[54] Castor T.P. (1998). Method and Apparatus for Isolating Therapeutic Compositions from Source Materials 1998. U.S. Patent.

[55] Keck G.E., Poudel Y.B., Cummins T.J., Rudra A., Covel J.A. (2010). Total Synthesis of Bryostatin 1. J. Am. Chem. Soc..

[56] Keck G.E., Truong A.P. (2005). Synthetic Studies on the Bryostatins: Preparation of a Truncated BC-Ring Intermediate by Pyran Annulation. Org. Lett..

[57] Keck G.E., Welch D.S., Poudel Y.B. (2006). Synthetic Studies toward Bryostatin 1: Preparation of a C1–C16 Fragment by Pyran Annulation. Tetrahedron Lett..

[58] Wender P.A., Hardman C.T., Ho S., Jeffreys M.S., Maclaren J.K., Quiroz R.V., Ryckbosch S.M., Shimizu A.J., Sloane J.L., Stevens M.C. (2017). Scalable Synthesis of Bryostatin 1 and Analogs, Adjuvant Leads against Latent HIV. Science.

[59] Tamao K., Sumitani K., Kumada M. (1972). Selective Carbon-Carbon Bond Formation by Cross-Coupling of Grignard Reagents with Organic Halides. Catal. By Nickel-Phosphine Complexes. J. Am. Chem. Soc..

[60] Corriu R.J.P., Masse J.P. (1972). Activation of Grignard Reagents by Transition-Metal Complexes. A New and Simple Synthesis of Trans-Stilbenes and Polyphenyls. J. Chem. Soc. Chem. Commun..

[61] Chu Z., Tong R., Yang Y., Song X., Hu T.B., Fan Y., Zhao C., Gao L., Song Z. (2021). Diverse Synthesis of the c Ring Fragment of Bryostatins via Zn/Cu-Promoted Conjugate Addition of α-Hydroxy Iodide with Enone. Chin. Chem. Lett..

[62] Rosse C., Linch M., Kermorgant S., Cameron A.J.M., Boeckeler K., Parker P.J. (2010). PKC and the Control of Localized Signal Dynamics. Nat. Rev. Mol. Cell Biol..

[63] Nishizuka Y. (1984). The Role of Protein Kinase c in Cell Surface Signal Transduction and Tumour Promotion. Nature.

[64] Li W., Zhu K., Liu Y., Liu M., Chen Q. (2025). Recent Advances in PKC Inhibitor Development: Structural Design Strategies and Therapeutic Applications. Eur. J. Med. Chem..

[65] Geraldes P., King G.L. (2010). Activation of Protein Kinase c Isoforms and Its Impact on Diabetic Complications. Circ. Res..

[66] Zhang D., Anantharam V., Kanthasamy A., Kanthasamy A.G. (2007). Neuroprotective Effect of Protein Kinase Cδ Inhibitor Rottlerin in Cell Culture and Animal Models of Parkinson’s Disease. J. Pharmacol. Exp. Ther..

[67] Isakov N. (2018). Protein Kinase c (PKC) Isoforms in Cancer, Tumor Promotion and Tumor Suppression. Semin. Cancer Biol..

[68] Marengo B., De Ciucis C., Ricciarelli R., Pronzato M.A., Marinari U.M., Domenicotti C. (2011). Protein Kinase C: An Attractive Target for Cancer Therapy. Cancers.

[69] Konopatskaya O., Poole A.W. (2010). Protein Kinase Cα: Disease Regulator and Therapeutic Target. Trends Pharmacol. Sci..

[70] Hirai H. (2018). Protein Kinase c in the Cerebellum: Its Significance and Remaining Conundrums. Cerebellum.

[71] Mochly-Rosen D., Das K., Grimes K.V. (2012). Protein Kinase C, an Elusive Therapeutic Target?. Nat. Rev. Drug Discov..

[72] Tian Z., Lu X.-T., Jiang X., Tian J. (2023). Bryostatin-1: A Promising Compound for Neurological Disorders. Front. Pharmacol..

[73] Yi P., Schrott L.M., Castor T., Alexander J. (2012). Bryostatin-1 vs. TPPB: Dose-Dependent APP Processing and PKC-α, -δ, and -ε Isoform Activation in SH-SY5Y Neuronal Cells. J. Mol. Neurosci..

[74] Nelson T.R., Cui C., Wang F., Alkon D.L. (2009). Reduction of β-Amyloid Levels by Novel Protein Kinase Cϵ Activators. J. Biol. Chem..

[75] Sun M.-K., Hongpaisan J., Nelson T., Alkon D. (2008). Poststroke Neuronal Rescue and Synaptogenesis Mediated in Vivo by Protein Kinase c in Adult Brains. Proc. Natl. Acad. Sci. USA.

[76] Farlow M., Thompson R., Wei L.-J., Tuchman A., Grenier E., Crockford D., Wilke S., Benison J., Alkon D. (2019). A Randomized, Double-Blind, Placebo-Controlled, Phase II Study Assessing Safety, Tolerability, and Efficacy of Bryostatin in the Treatment of Moderately Severe to Severe Alzheimer’s Disease. J. Alzheimer’s Dis. JAD.

[77] Thomas A., Pepper C., Hoy T., Bentley P. (2004). Bryostatin Induces Protein Kinase c Modulation, Mcl-1 Up-Regulation and Phosphorylation of Bcl-2 Resulting in Cellular Differentiation and Resistance to Drug-Induced Apoptosis in B-Cell Chronic Lymphocytic Leukemia Cells. Leuk. Lymphoma.

[78] Tan Y., Ruan H., Demeter M.R., Comb M.J. (1999). P90RSK Blocks Bad-Mediated Cell Death via a Protein Kinase C-Dependent Pathway. J. Biol. Chem..

[79] Ruvolo P.P., Deng X., Carr B.K., May W.S. (1998). A Functional Role for Mitochondrial Protein Kinase Cα in Bcl2 Phosphorylation and Suppression of Apoptosis. J. Biol. Chem..

[80] al-Katib A., Mohammad R.M., Dan M., Hussein M.E., Akhtar A., Pettit G.R., Sensenbrenner L.L. (1993). Bryostatin 1-Induced Hairy Cell Features on Chronic Lymphocytic Leukemia Cells in Vitro. Exp. Hematol..

[81] Mohammad R.M., Beck F.W.J., Katato K., Hamdy N., Wall N., Al-Katib A. (1998). Potentiation of 2-Chlorodeoxyadenosine Activity by Bryostatin 1 in the Resistant Chronic Lymphocytic Leukemia Cell Line (WSU-CLL): Association with Increased Ratios of DCK/5′-NT and Bax/Bcl-2. Biol. Chem..

[82] Kitada S., Zapata J.M., Andreeff M., Reed J.C. (1999). Bryostatin and CD40-Ligand Enhance Apoptosis Resistance and Induce Expression of Cell Survival Genes in B-Cell Chronic Lymphocytic Leukaemia. Br. J. Haematol..

[83] Asiedu C., Biggs J., Lilly M., Kraft A.S. (1995). Inhibition of Leukemic Cell Growth by the Protein Kinase c Activator Bryostatin 1 Correlates with the Dephosphorylation of Cyclin-Dependent Kinase 2. Cancer Res..

[84] Vrana J.A., Saunders A.M., Chellappan S.P., Grant S. (1998). Divergent Effects of Bryostatin 1 and Phorbol Myristate Acetate on Cell Cycle Arrest and Maturation in Human Myelomonocytic Leukemia Cells (U937). Differentiation.

[85] Vrana J.A., Kramer L.B., Saunders A.M., Zhang X.-F., Dent P., Povirk L.F., Grant S. (1999). Inhibition of Protein Kinase c Activator-Mediated Induction of P21CIP1 and P27KIP1 by Deoxycytidine Analogs in Human Leukemia Cells. Biochem. Pharmacol..

[86] Rajabi S., Maresca M., Yumashev A.V., Choopani R., Hajimehdipoor H. (2021). The Most Competent Plant-Derived Natural Products for Targeting Apoptosis in Cancer Therapy. Biomolecules.

[87] Dalisay D.S., Tenebro C.P., Sabido E.M., Faith A., June M., Reyes-Salarda R., Saludes J.P. (2024). Marine-Derived Anticancer Agents Targeting Apoptotic Pathways: Exploring the Depths for Novel Cancer Therapies. Mar. Drugs.

[88] Deep A., Kumar D., Bansal N., Narasimhan B., Marwaha R.K., Sharma P.C. (2023). Understanding Mechanistic Aspects and Therapeutic Potential of Natural Substances as Anticancer Agents. Phytomed. Plus.

[89] Wang Z., Wang S., Dai Y., Grant S. (2002). Bryostatin 1 Increases 1-β-d-Arabinofuranosylcytosine-Induced Cytochrome c Release and Apoptosis in Human Leukemia Cells Ectopically Expressing Bcl-XL. J. Pharmacol. Exp. Ther..

[90] Wall N.R., Mohammad R.M., Reddy K.B., Al-Katib A.M. (2000). Bryostatin 1 Induces Ubiquitination and Proteasome Degradation of Bcl-2 in the Human Acute Lymphoblastic Leukemia Cell Line, Reh. Int. J. Mol. Med..

[91] Vrana J.A., Grant S. (2001). Synergistic Induction of Apoptosis in Human Leukemia Cells (U937) Exposed to Bryostatin 1 and the Proteasome Inhibitor Lactacystin Involves Dysregulation of the PKC/MAPK Cascade. Blood.

[92] Wang S., Wang Z., Boise L., Dent P., Grant S. (1999). Bryostatin 1 Enhances Paclitaxel-Induced Mitochondrial Dysfunction and Apoptosis in Human Leukemia Cells (U937) Ectopically Expressing Bcl-XL. Leukemia.

[93] Von Burstin V.A., Xiao L., Kazanietz M.G. (2010). Bryostatin 1 Inhibits Phorbol Ester-Induced Apoptosis in Prostate Cancer Cells by Differentially Modulating Protein Kinase c (PKC) δ Translocation and Preventing PKCδ-Mediated Release of Tumor Necrosis Factor-α. Mol. Pharmacol..

[94] Stang S.L., Lopez-Campistrous A., Song X., Dower N.A., Blumberg P.M., Wender P.A., Stone J.C. (2009). A Proapoptotic Signaling Pathway Involving RasGRP, Erk, and Bim in B Cells. Exp. Hematol..

[95] Zhang X., Zhang R., Zhao H., Cai H., Gush K.A., Kerr R.G., Pettit G.R., Kraft A.S. (1996). Preclinical Pharmacology of the Natural Product Anticancer Agent Bryostatin 1, an Activator of Protein Kinase C. Cancer Res..

[96] Khan P., McGown A.T., Dawson M.J., Jayson G., Prendiville J.A., Pettit G.R., Crowther D. (1998). High-Performance Liquid Chromatographic Assay for the Novel Antitumor Drug, Bryostatin-1, Incorporating a Serum Extraction Technique. J. Chromatogr. B Biomed. Sci. Appl..

[97] Zhao M., Rudek M.A., He P., Smith B.D., Baker S.D. (2005). Validation and Implementation of a Method for Determination of Bryostatin 1 in Human Plasma by Using Liquid Chromatography/Tandem Mass Spectrometry. Anal. Biochem..

[98] Korecka M., Shaw L.M. (2009). Review of the Newest HPLC Methods with Mass Spectrometry Detection for Determination of Immunosuppressive Drugs in Clinical Practice. Ann. Transplant..

[99] McGown A.T., Jayson G., Pettit G.R., Haran M.S., Ward T.H., Crowther D. (1998). Bryostatin 1-Tamoxifen Combinations Show Synergistic Effects on the Inhibition of Growth of P388 Cells in Vitro. Br. J. Cancer.

[100] Biberacher V., Decker T., Oelsner M., Wagner M., Bogner C., Schmidt B., Kreitman R.J., Peschel C., Pastan I., Meyer C. (2012). The Cytotoxicity of Anti-CD22 Immunotoxin Is Enhanced by Bryostatin 1 in B-Cell Lymphomas through CD22 Upregulation and PKC-βII Depletion. Haematologica.

[101] Koutcher J.A., Motwani M., Zakian K.L., Li X.K., Matei C., Dyke J.P., Ballon D., Yoo H.H., Schwartz G.K. (2000). The in Vivo Effect of Bryostatin-1 on Paclitaxel-Induced Tumor Growth, Mitotic Entry, and Blood Flow. Clin. Cancer Res..

[102] Mohammad R.M., Varterasian M.L., Almatchy V.P., Hannoudi G.N., Pettit G., Al-Katib A. (1998). Successful Treatment of Human Chronic Lymphocytic Leukemia Xenografts with Combination Biological Agents Auristatin PE and Bryostatin 1. Clin. Cancer Res..

[103] Mohanty S., Huang J., Basu A. (2005). Enhancement of Cisplatin Sensitivity of Cisplatin-Resistant Human Cervical Carcinoma Cells by Bryostatin 1. Clin. Cancer Res..

[104] Szallasi Z., Smith C.B., Pettit G.R., Blumberg P.M. (1994). Differential Regulation of Protein Kinase c Isozymes by Bryostatin 1 and Phorbol 12-Myristate 13-Acetate in NIH 3T3 Fibroblasts. J. Biol. Chem..

[105] Yan W., Chen W.C., Liu Z., Huang L. (2010). Bryostatin-I: A Dendritic Cell Stimulator for Chemokines Induction and a Promising Adjuvant for a Peptide Based Cancer Vaccine. Cytokine.

[106] Schuchter L.M., Esa A.H., May S., Laulis M.K., Pettit G.R., Hess A.D. (1991). Successful Treatment of Murine Melanoma with Bryostatin 1. Cancer Res..

[107] Mohammad R.M., Wall N.R., Dutcher J.A., Al-Katib A.M. (2000). The Addition of Bryostatin 1 to Cyclophosphamide, Doxorubicin, Vincristine, and Prednisone (CHOP) Chemotherapy Improves Response in a CHOP-Resistant Human Diffuse Large Cell Lymphoma Xenograft Model. Clin. Cancer Res..

[108] Erin N., Tavşan E., Akdeniz Ö., Isca V.M.S., Rijo P. (2021). Rebound Increases in Chemokines by CXCR2 Antagonist in Breast Cancer Can Be Prevented by PKCδ and PKCε Activators. Cytokine.

[109] Drexler H.G., Gignac S.M., Jones R.A., Scott C.S., Pettit G.R., Hoffbrand A.V. (1989). Bryostatin 1 Induces Differentiation of B-Chronic Lymphocytic Leukemia Cells. Blood.

[110] Jones R.J., Sharkis S.J., Miller C.B., Rowinsky E.K., Burke P.J., May W.S. (1990). Bryostatin 1, a Unique Biologic Response Modifier: Anti-Leukemic Activity in Vitro. Blood.

[111] Scheid C., Prendiville J., Jayson G., Crowther D., Fox B., Pettit G.R., Stern P.L. (1994). Immunomodulation in Patients Receiving Intravenous Bryostatin 1 in a Phase I Clinical Study: Comparison with Effects of Bryostatin 1 on Lymphocyte Function in Vitro. Cancer Immunol. Immunother..

[112] Ariza M.E., Ramakrishnan R., Singh N.P., Chauhan A., Nagarkatti P.S., Nagarkatti M. (2010). Bryostatin-1, a Naturally Occurring Antineoplastic Agent, Acts as a Toll-like Receptor 4 (TLR-4) Ligand and Induces Unique Cytokines and Chemokines in Dendritic Cells. J. Biol. Chem..

[113] Spitaler M., Utz I., Hilbe W., Hofmann J., Grunicke H.H. (1998). PKC-Independent Modulation of Multidrug Resistance in Cells with Mutant (V185) but Not Wild-Type (G185) P-Glycoprotein by Bryostatin 1. Biochem. Pharmacol..

[114] Al-Katib A.M., Smith M.R., Kamanda W.S., Pettit G.R., Hamdan M., Mohamed A.N., Chelladurai B., Mohammad R.M. (1998). Bryostatin 1 Down-Regulates Mdr1 and Potentiates Vincristine Cytotoxicity in Diffuse Large Cell Lymphoma Xenografts. Clin. Cancer Res..

[115] Wang H., Mohammad R.M., Werdell J., Shekhar P.V. (1998). P53 and Protein Kinase c Independent Induction of Growth Arrest and Apoptosis by Bryostatin 1 in a Highly Metastatic Mammary Epithelial Cell Line: In Vitro versus in Vivo Activity. Int. J. Mol. Med..

[116] Wang J., Wang Z., Sun Y., Liu D. (2019). Bryostatin-1 Inhibits Cell Proliferation of Hepatocarcinoma and Induces Cell Cycle Arrest by Activation of GSK3β. Biochem. Biophys. Res. Commun..

[117] Zeng N., Xu Y., Wu Y., Hongbo T., Wu M. (2017). Bryostatin 1 Causes Attenuation of TPA-Mediated Tumor Promotion in Mouse Skin. Mol. Med. Rep..

[118] Dowlati A., Lazarus H.M., Hartman P., Jacobberger J.W., Whitacre C., Gerson S.L., Ksenich P., Cooper B.W., Frisa P.S., Gottlieb M. (2003). Phase I and Correlative Study of Combination Bryostatin 1 and Vincristine in Relapsed B-Cell Malignancies. Clin. Cancer Res. Off. J. Am. Assoc. Cancer Res..

[119] Roberts J.D., Smith M.R., Feldman E.J., Cragg L., Millenson M.M., Roboz G.J., Honeycutt C., Thune R., Padavic-Shaller K., Carter W.H. (2006). Phase I Study of Bryostatin 1 and Fludarabine in Patients with Chronic Lymphocytic Leukemia and Indolent (Non-Hodgkin’s) Lymphoma. Clin. Cancer Res..

[120] Haas N.B., Smith M., Lewis N., Littman L., Yeslow G., Joshi I.D., Murgo A., Bradley J., Gordon R., Wang H. (2003). Weekly Bryostatin-1 in Metastatic Renal Cell Carcinoma: A Phase II Study. Clin. Cancer Res..

[121] Prendiville J., Crowther D., Thatcher N., Woll P.J., Fox B.W., McGown A., Testa N., Stern P., McDermott R., Potter M. (1993). A Phase I Study of Intravenous Bryostatin 1 in Patients with Advanced Cancer. Br. J. Cancer.

[122] Philip P.A., Rea D., Thavasu P., Carmichael J., Stuart N.S., Rockett H., Talbot D.C., Ganesan T., Pettit G.R., Balkwill F. (1993). Phase I Study of Bryostatin 1: Assessment of Interleukin 6 and Tumor Necrosis Factor Induction in Vivo. J. Natl. Cancer Inst..

[123] Ahmad I., Al-Katib A.M., Beck F.W., Mohammad R.M. (2000). Sequential Treatment of a Resistant Chronic Lymphocytic Leukemia Patient with Bryostatin 1 Followed by 2-Chlorodeoxyadenosine: Case Report. Clin. Cancer Res..

[124] Zonder J.A., Shields A.F., Zalupski M., Chaplen R., Heilbrun L.K., Arlauskas P., Philip P.A. (2001). A Phase II Trial of Bryostatin 1 in the Treatment of Metastatic Colorectal Cancer1. Clin. Cancer Res..

[125] Tozer R.G., Burdette-Radoux S., Berlanger K., Davis M.L., Lohmann R.C., Rusthoven J.R., Wainman N., Zee B., Seymour L. (2002). A Randomized Phase II Study of Two Schedules of Bryostatin-1 (NSC339555) in Patients with Advanced Malignant Melanoma—A National Cancer Institute of Canada Clinical Trials Group Study. Investig. New Drugs.

[126] Varterasian M.L., Mohammad R.M., Eilender D.S., Hulburd K., Rodriguez D.H., Pemberton P.A., Pluda J.M., Dan M.D., Pettit G.R., Chen B.D. (1998). Phase I Study of Bryostatin 1 in Patients with Relapsed Non-Hodgkin’s Lymphoma and Chronic Lymphocytic Leukemia. J. Clin. Oncol..

[127] Jayson G.C., Crowther D., Prendiville J.A., McGown A.T., Scheid C., Stern P.L., Young R., Brenchley P., Chang J., Owens S.M. (1995). A Phase I Trial of Bryostatin 1 in Patients with Advanced Malignancy Using a 24 Hour Intravenous Infusion. Br. J. Cancer.

[128] Varterasian M.L., Mohammad R.M., Shurafa M.S., Hulburd K., Pemberton P.A., Rodriguez D.H., Spadoni V., Eilender D.S., Murgo A., Wall N. (2000). Phase II Trial of Bryostatin 1 in Patients with Relapsed Low-Grade Non-Hodgkin’s Lymphoma and Chronic Lymphocytic Leukemia. Clin. Cancer Res..

[129] El-Rayes B.F., Gadgeel S., Shields A.F., Manza S., Lorusso P., Philip P.A. (2006). Phase I Study of Bryostatin 1 and Gemcitabine. Clin. Cancer Res..

[130] Propper D.J., Macaulay V., O’Byrne K.J., Braybrooke J.P., Wilner S.M., Ganesan T.S., Talbot D.C., Harris A.L. (1998). A Phase II Study of Bryostatin 1 in Metastatic Malignant Melanoma. Br. J. Cancer.

[131] Cragg L.H., Andreeff M., Feldman E., Roberts J., Murgo A., Winning M., Tombes M.B., Roboz G., Kramer L., Grant S. (2002). Phase I Trial and Correlative Laboratory Studies of Bryostatin 1 (NSC 339555) and High-Dose 1-B-D-Arabinofuranosylcytosine in Patients with Refractory Acute Leukemia. Clin. Cancer Res..

[132] Grant S., Roberts J., Poplin E., Tombes M.B., Kyle B., Welch D., Carr M., Bear H.D. (1998). Phase Ib Trial of Bryostatin 1 in Patients with Refractory Malignancies. Clin. Cancer Res..

[133] Morgan R.J., Leong L., Chow W., Gandara D., Frankel P., Garcia A., Lenz H.-J., Doroshow J.H. (2010). Phase II Trial of Bryostatin-1 in Combination with Cisplatin in Patients with Recurrent or Persistent Epithelial Ovarian Cancer: A California Cancer Consortium Study. Investig. New Drugs.

